# Differences in configural processing for human versus android dynamic facial expressions

**DOI:** 10.1038/s41598-023-44140-4

**Published:** 2023-10-07

**Authors:** Alexander Diel, Wataru Sato, Chun-Ting Hsu, Takashi Minato

**Affiliations:** 1 RIKEN Information R&D and Strategy Headquarters, Guardian Robot Project, Kyoto, Japan; 2 School of Psychology, Cardiff University, Cardiff, UK

**Keywords:** Psychology, Human behaviour

## Abstract

Humanlike androids can function as social agents in social situations and in experimental research. While some androids can imitate facial emotion expressions, it is unclear whether their expressions tap the same processing mechanisms utilized in human expression processing, for example configural processing. In this study, the effects of global inversion and asynchrony between facial features as configuration manipulations were compared in android and human dynamic emotion expressions. Seventy-five participants rated (1) angry and happy emotion recognition and (2) arousal and valence ratings of upright or inverted, synchronous or asynchronous, android or human agent dynamic emotion expressions. Asynchrony in dynamic expressions significantly decreased all ratings (except valence in angry expressions) in all human expressions, but did not affect android expressions. Inversion did not affect any measures regardless of agent type. These results suggest that dynamic facial expressions are processed in a synchrony-based configural manner for humans, but not for androids.

## Introduction

Android robots are unique artificial agents that can imitate humanlike emotional facial expressions, which could be beneficial both for research and social applications. For research use, androids can be an attractive alternative in facial expression research for real-life interactions with realistic yet well-controlled situations to investigate the processing of facial expressions^1^. For applications, androids can perform social support functions in elderly care, service, education, and emotional labour^2–6^. However, several issues exist on androids’ abilities to replicate human emotional expressions. Facial expressions can be defined by specific sets of facial muscle movements^7,8^, and thus social androids should be able to reliably imitate said movement sets. Statistical evaluation of androids’ abilities to expression face emotions are lacking and are focused on a limited number of emotional expressions^9^.

A recent study has developed and validated an android, called Nikola, that can show facial expressions of six basic emotions like humans using pneumatic actuators with a temporal resolution of milliseconds^9^. In said study, the android Nikola could show recognizable basic emotions of anger, disgust, fear, happiness, sadness, and surprise. The study replicated results from previous research on the effects of dynamic expression speed (of face action unit motion) on emotion recognition using Nikola’s expressions.

However, it remains unknown whether the emotional facial expressions of humans and androids could be processed in a similar mode. Ample psychological evidence has indicated that human facial structure and expressions are processed in a configural manner^10–13^. To the best of our knowledge, no study investigated whether the facial expressions of an android like Nikola could be similarly processed in the configural mode.

To investigate this issue, we used two methods to impair the configural processing for facial expressions: Inversion and asynchrony manipulation.

Previous studies have shown that the inverted presentations of dynamic expressions disrupt emotion recognition^10–12^. Studies testing basic face perception indicated that inverted presentations impair face processing due to the difficulty associated with configural or holistic effects^14,15^. For example, Farah et al.^14^ instructed participants to process faces either holistically or partially and asked them to recognize faces presented in an upright or inverted position. The results showed that inversion impaired the recognition of faces only when subjects processed faces holistically. An upright recognition advantage can be explained with an increased configural or holistic processing developed through extensive experience with upright (but not inverted) faces^16^. Similarly, research showing an upright recognition advantage for dynamic expressions indicates a role of configural processing in facial expressions. As android expression processing is supposed to approximate human expression processing, inversion effects for android emotion recognition are here expected.

The second method is to manipulate asynchrony between facial features. The processing of synchronous and asynchronous expressions recruits activity in different brain centres, potentially reflecting global versus local processing of face expression information^17^. Furthermore, synchronous motion helps bind motion features in facial expressions which is disrupted when facial muscles move in an asynchronous manner^18^. Thus, an asynchrony manipulation can disrupt the configural processing of dynamic emotional expressions.

To investigate the role of configural processing in an androids’ emotional expression, we presented videos of angry and happy dynamic facial expressions of humans and Nikola in the upright vs. inverted direction and synchronous (normal) vs. asynchronous mode. If configural processing between a human and an android is analogous, then inversion and asynchrony should both disrupt the emotion recognition of human and android agents. To further investigate the effects on emotion processing, valence (pleasure–displeasure) and arousal (physiological excitation) dimensional measures from the circumplex model, a widely used model of the assessment of facial emotion expressions, was used^19^.

The hypotheses were as follows:

1. Inversion reduces the ability to recognize angry and happy expressions for both human and android agent expressions.
2. Asynchrony reduces the ability to recognize angry and happy expressions for both human and agent expressions.
3. Inversion reduces valence and arousal ratings in human and agent expressions.
4. Asynchrony reduces valence and arousal ratings in human and agent expressions.

## Results

### Emotion recognition

**Figure 1:**
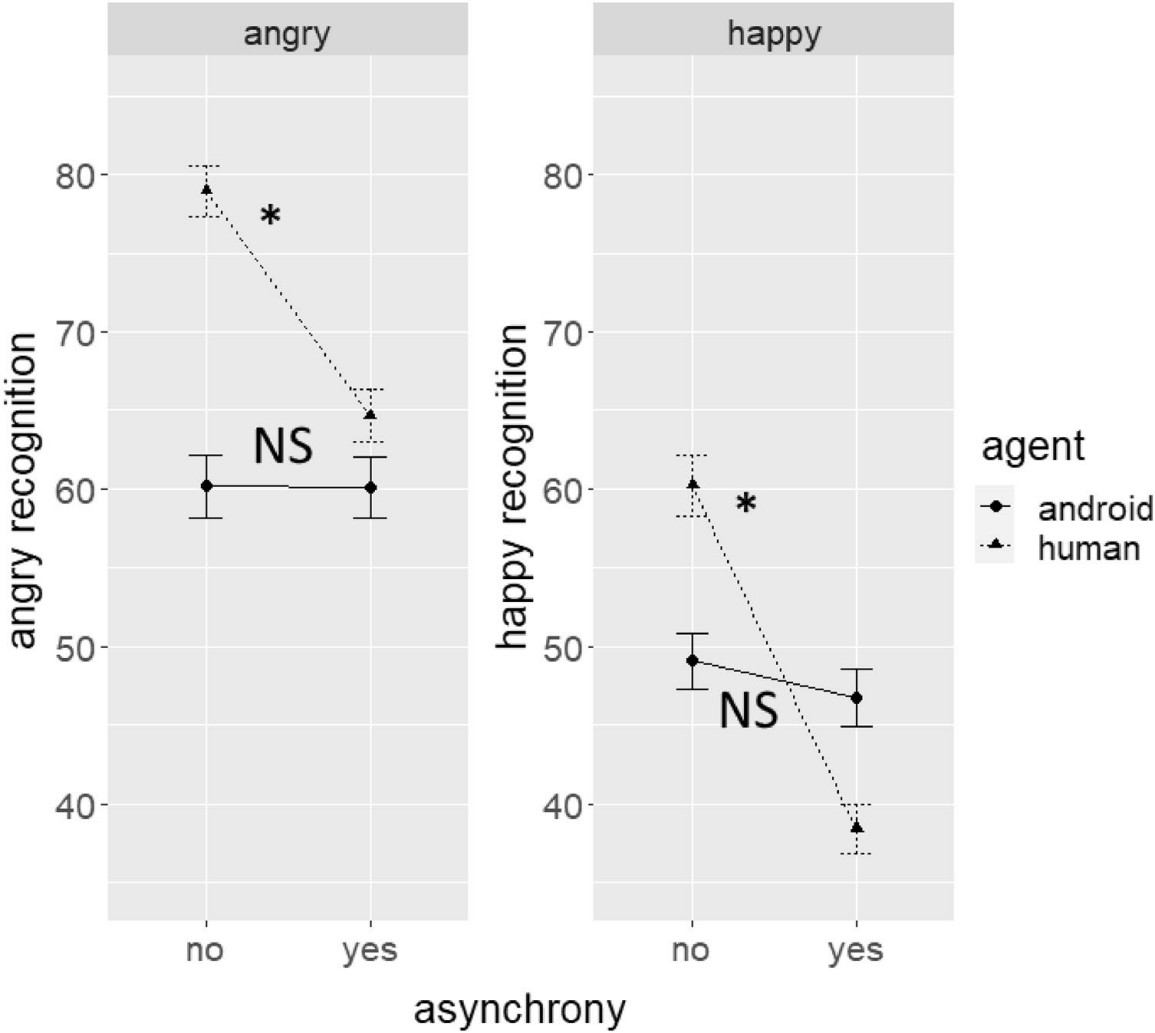
Mean (with standard error) target emotion ratings divided by asynchrony and agent conditions. Error bars indicate standard errors and asterisks show significant differences (which were found for human agents only).

Within-subject ANOVAs were conducted on the ratings of angry and happy recognition scales with orientation, asynchrony, agent, and emotion as factors. Results for both angry and happy ratings are depicted in Fig. 1.

For the recognition of anger, significant main effects were observed for orientation (*F*(1,1163) = 5.3, p = 0.022, *η*_p_^2^ = 0.004) and emotion (*F*(1, 1163) = 1584.63, *p* < 0.001, *η*_p_^2^ = 0.58), and significant interactions between agent and emotion (F(1,1163) = 8.33, *p* = 0.003, *η*_p_^2^ = 0.007), asynchrony and emotion (*F*(1,1163) = 19.83, *p* < 0.001, *η*_p_^2^ = 0.02), and asynchrony, agent, and emotion (*F*(1,1163) = 18.57, *p* < 0.001, *η*_p_^2^ = 0.02). Orientation effects for angry and happy emotions are depicted in Figure A1.

For the main effect of orientation, however, the follow-up Bonferroni-corrected Tukey tests showed no significant differences between upright and inverted expressions (*t*(1111) = 1.44, *p*_adj_ = 0.151).

For the interaction between asynchrony, agent, and emotion, Bonferroni-corrected Tukey-tests on the effect of asynchrony were analysed for each agent's target emotion condition. Asynchrony decreased emotion recognition of angry human expressions (*t*(1105) = 6.16, *p*_adj_ < 0.001), but not for angry android expressions (*t*(1105) = 0.01, *p*_adj_ = 1) expressions.

For the recognition of happiness, the results showed significant main effects for orientation (*F*(1,1163) = 5.55, *p* = 0.019, *η*_p_^2^ = 0.005), asynchrony (*F*(1,1163) = 11.71., *p* < 0.001, *η*_p_^2^ = 0.01), emotion (*F*(1,1163) = 1160.14, *p* < 0.001, *η*_p_^2^ = 0.05), and significant interactions between asynchrony and agent (*F*(1,1163) = 6.47, *p* = 0.038, *η*_p_^2^ = 0.004), agent and emotion (*F*(1,1163) = 33.98, *p* < 0.011, *η*_p_^2^ = 0.006), asynchrony and emotion (*F*(1,1163) = 33.98, *p* < 0.001, *η*_p_^2^ = 0.03), and asynchrony, agent, and emotion (*F*(1,1163) = 28.23, *p* < 0.001, *η*_p_^2^ = 0.02).

The patterns of post-hoc Bonferroni-corrected Tukey tests were identical to that of angry expressions. For the main effect of orientation, no differences between orientation conditions were found (*t*(1111) = − 0.7, *p*_adj_ = 0.48). For the interaction between asynchrony, agent, and emotion, the tests of asynchrony showed that asynchrony reduced happy recognition for happy human (*t*(1105) = 10.95, *p*_adj_ < 0.001), but not happy android (*t*(1105) = 1.18, *p*_adj_ = 0.478) expressions.

In summary, asynchrony meanwhile decreased the ability to correctly recognize human expressions, but not for androids. No inversion effects were observed.

### Valence and arousal

Within-subject ANOVAs were conducted on valence and arousal ratings with orientation, asynchrony, agent, and emotion as factors.

**Figure 2:**
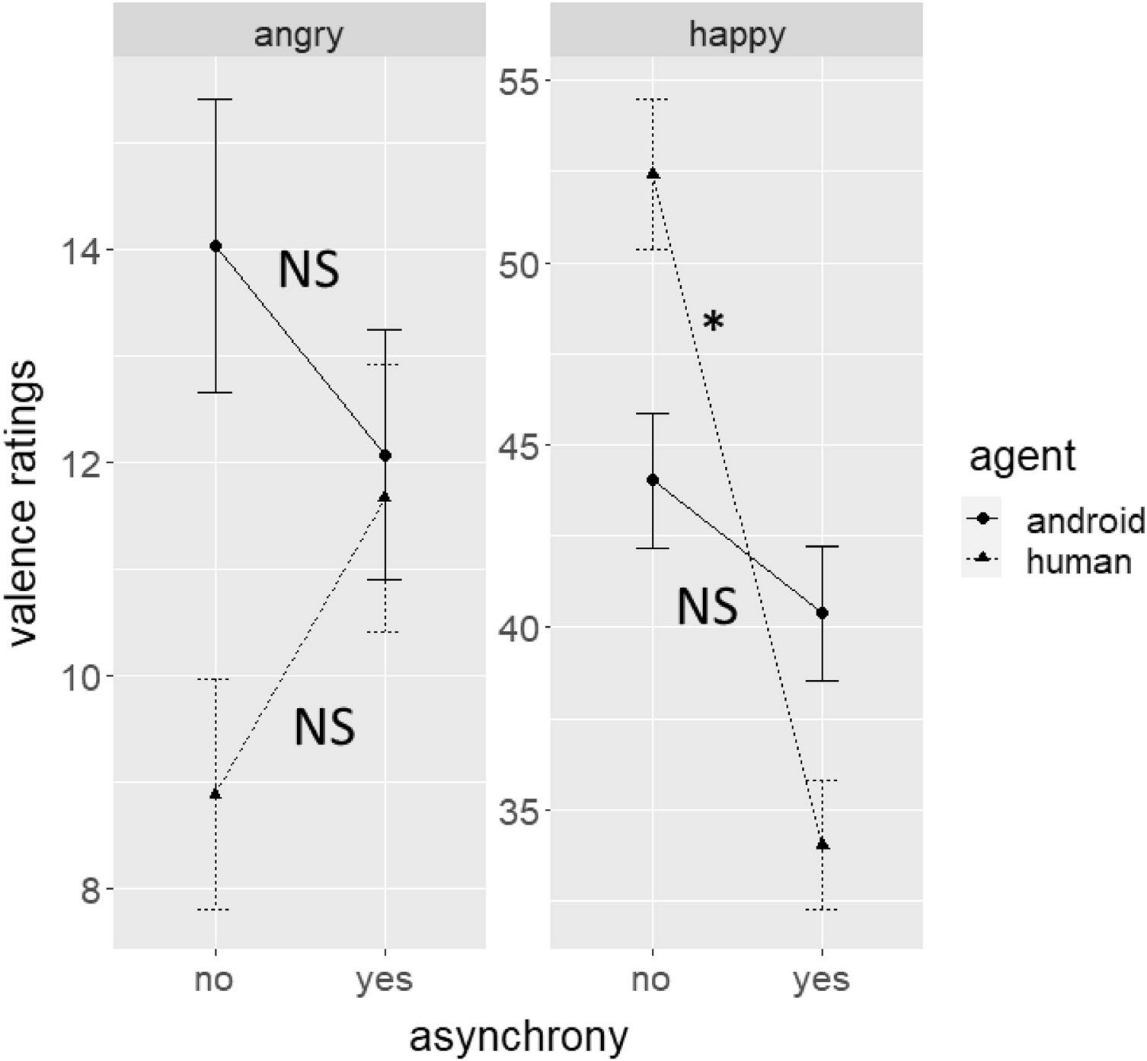
Average valence ratings divided by emotion, agent, and asynchrony conditions. Error bars indicate standard errors and asterisks show significant differences (which were only found for happy human expressions).

For valence ratings (Fig. 2), the results showed significant main effects for asynchrony (*F*(1,1163) = 5.76, *p* = 0.017, *η*_p_^2^ = 0.005) and emotion (*F*(1,1163) = 605.63, *p* < 0.001, *η*_p_^2^ = 0.34), and significant interactions between asynchrony and emotions (*F*(1,1163) = 21.47, *p* < 0.001, *η*_p_^2^ = 0.02) and asynchrony, agent, and emotion (*F*(1,1163) = 16.35, *p* < 0.001 , *η*_p_^2^ = 0.01).

For the interaction between asynchrony, agent, and emotion, post-hoc Bonferroni-corrected Tukey tests on the effect of asynchrony were analysed for each of the agent × emotion conditions. The results revealed that asynchrony decreased valence ratings in happy human expressions (*t*(1105) = 8.47, *p*_adj_ < 0.001), but not in angry human (*t*(1105) = 1.33, *p*_adj_ = 1), angry android (*t*(1105) = 0.9, *p*_adj_ = 1), or happy android expressions (*t*(1105) = 1.67, *p*_adj_ = 1).

**Figure 3:**
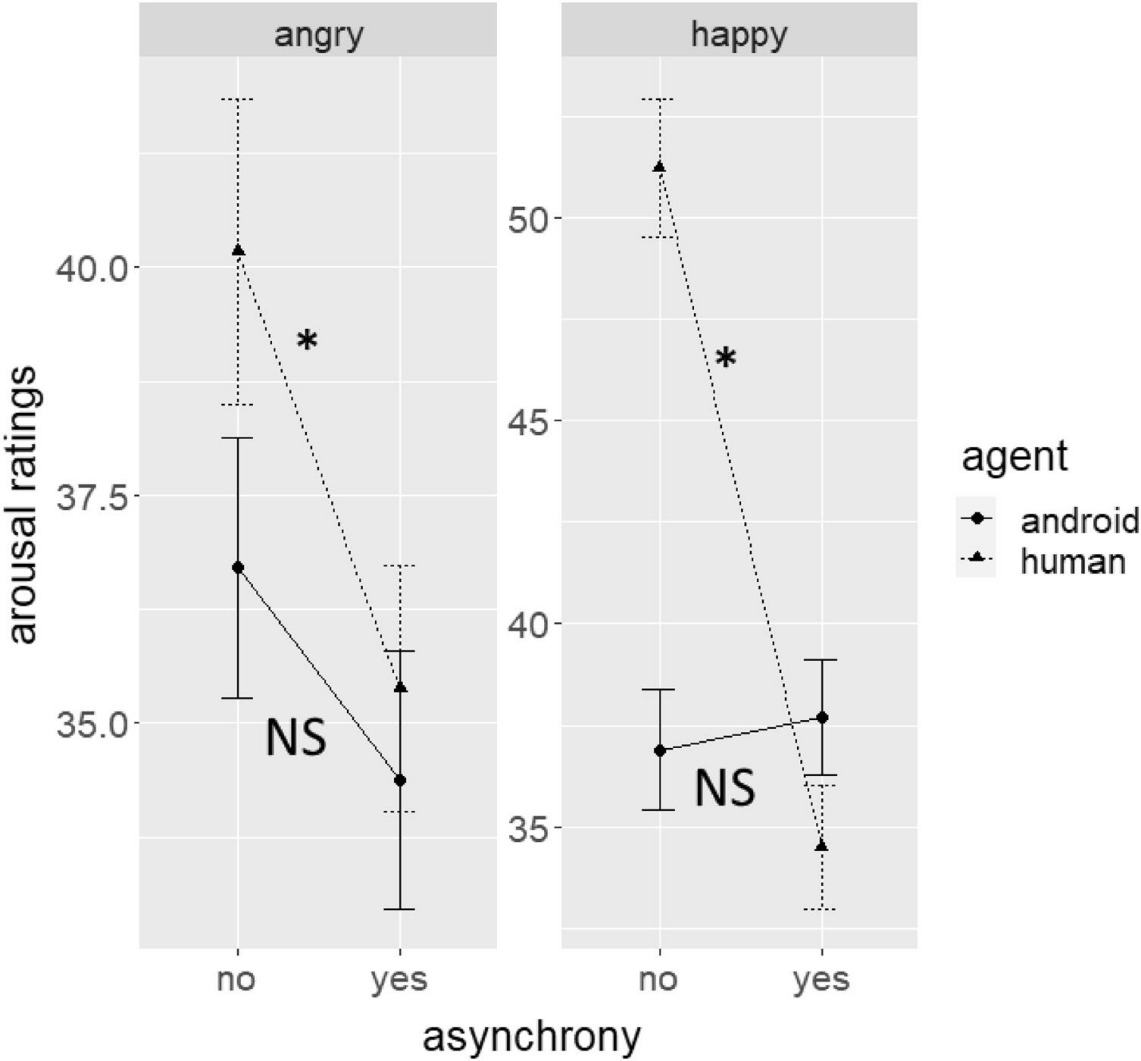
Average arousal ratings divided by emotion, agent, and asynchrony conditions. Error bars indicate standard errors and asterisks show significant differences (which were present only for human agents).

For arousal ratings (Fig. 3), the results showed significant main effects for asynchrony (*F*(1,1163) = 8.7, *p* = 0.003, *η*_p_^2^ = 0.007) and emotion (*F*(1,1163) = 5.71, *p* = 0.017, *η*_p_^2^ = 0.005), and significant interactions between asynchrony and agent (*F*(1,1163) = 4.93, *p* = 0.027, *η*_p_^2^ = 0.004) and asynchrony, agent, and emotion (*F*(1,1163) = 7.23, *p* = 0.007, *η*_p_^2^ = 0.006).

Bonferroni-adjusted Post-hoc Tukey tests for the interaction between asynchrony, agent, and emotion revealed that asynchrony significantly reduced arousal ratings in angry human (*t*(1105) = 2.29, *p*_adj_ = 0.044) and happy human expressions (*t*(1105) = 8.2, *p*_adj_ < 0.001), but not in angry android (*t*(1105) = 1.14, *p*_adj_ = 0.51) or happy android expressions (*t*(1105) = 0.4, *p*_adj_ = 1).

In summary, the effect of asynchrony on valence and arousal differed again between agents: While asynchrony decreased valence and arousal ratings for human expressions, it did not affect any of the android’s expressions. Again, evidence for an inversion effect was not found.

## Discussion

The study’s goal was to investigate the effects of inversion and asynchrony of the processing of emotions in human and android expressions. Contrary to previous research, inversion did not affect emotion recognition in either android or human agents. Meanwhile, asynchrony reduced the ability to correctly recognize angry and happy expressions in human faces while it did not affect android faces. Furthermore, arousal and valence ratings only decreased for angry and happy human (not android) faces and no effects of inversion were observed. Thus, hypotheses 1 and 3 (inversion effects) were not supported and hypotheses 2 and 4 (asynchrony effects) were only observed for human but not android expressions. Previous research found that inversion reduces the ability to recognize emotions in dynamic facial expressions^10–12^. Meanwhile, inversion effects on arousal and valence ratings have been more mixed^20–22^. However, in this study no inversion effect on any variable has been observed. It is possible that due to the limited range of emotional expressions present in this study (angry and happy) and because expressions could be watched indefinitely, participants could rely more on feature-based expression recognition in inverted conditions. Alternatively, certain features present in the expressions (e.g., an open mouth in happy faces) may have facilitated feature-based processing^23^. In fact, inversion effects are not consistently found for emotion expression recognition and especially not for happy expressions^24–26^ which were used in this study.

Asynchrony decreased both emotion recognition and arousal and valence ratings for human expressions, indicating that asynchrony disrupts the typical processing of human emotional expressions. Interestingly, asynchrony did not affect emotion processing in android expressions. One possibility is that featural processing is increased for android expressions and thus the observation of individual AU motions, rather than the synchrony of the whole expression, is sufficient to recognize the emotion. However, as no inversion effects were observed, this agent effect cannot be explained by differences in configural processing.

Alternatively, participants may be more sensitive to asynchronies in real human compared to android faces. Thus, the same level of asynchrony may have stronger effects on human compared to android expressions: Face-related processing decreased for robot and android faces compared to human faces^27,28^. However, both previous studies also used mechanical-looking faces rather than realistic faces including humanlike faces such as Nikola’s—hence, it is unclear whether this decreased face-related processing in android faces can be applied to this study. Asynchronies can disrupt configural processing in facial expressions^17,18^. However, no effects of inversion have been observed in this study, complicating interpretations of the involvement of configural processing. Furthermore, even though the instructions asked to observe the stimuli the way they were presented, participants may have turned their heads for inverted expressions, thus negating orientation effects. However, online experiments find effects of inversion in face rating tasks^32^, indicating that participants generally do not tend to rotate their screens for inverted stimuli when not supervised. Finally, response times were not measured in this experiment. As delayed response times may indicate difficulty and uncertainty, response time analysis may provide an additional indicator of disturbed emotion recognition processing when used in future research.

Previous research on asynchrony or inversion used computer-generated (CG) face stimuli^17,18^ while this study is the first to investigate the role of asynchrony in human expressions. CG faces recruit decreased levels of configural processing, and human responses to CG emotion expressions tend to be impoverished compared to human expressions^29,30^. A decreased level of emotion processing in CG faces may not survive global inversion, thus diminishing asynchrony-related processing. Meanwhile, deeper processing of human facial expressions may allow the processing to remain present even when stimuli are inverted.

## Methods

### Participants

Seventy-five Japanese participants (36 female, 37 male, 2 not reported; age, *M* = 30.85, SD = 4.3, and ranged from 18 to 35) were recruited via CrowdWorks (Tokyo, Japan). The sample size was determined via a-priori power analysis. We assumed to conduct a 2 × 2 × 2 × 2 repeated-measures analysis of variance (ANOVA) with an α of 0.05, power (1 – β) of 0.80, effect size f of 0.10 (weak), and correlation among repeated measures of 0.5. The results showed that more than 60 participants were needed. All participants provided informed consent before participating in the study. The study was approved by the RIKEN Ethics Committee and performed in accordance with the Declaration of Helsinki.

### Materials

Video clips (1.25 s each) of emotion expressions were used, divided by two agents (android, human), two emotions (angry, happy), two orientations (upright, inverted), and two asynchrony levels (synchronous, asynchronous). In the asynchrony conditions, the upper right half of the face moved with a 500 ms delay, and the upper left half with a 1000 ms delay starting after motion onset. Human videos were created from the AIST Expression Database^31^ and asynchronies were created using the cropping tool of the Adobe Premiere video editing software. Android videos were created by filming the front face of the android Nikola while it expressed angry and happy emotions and asynchronies were created by delaying the programmed motion onset of the relevant actuators. Actuators (which imitate specific face AUs) were chosen according to previous research on Nikola’s empirically validated basic emotions^9^. Specifically, for angry expressions, the following AUs were used: 4 (brow lowerer), 5 (upper lid raiser), 7 (lid tightener), 23 (lip tightener), and 25 (lips part). For happy expressions, the following AUs were used: 1 (inner row raiser), 6 (cheek raiser), 12 (lip corner puller), 15 (lip corner depressor), and 25 (lips part).

**Figure 4:**
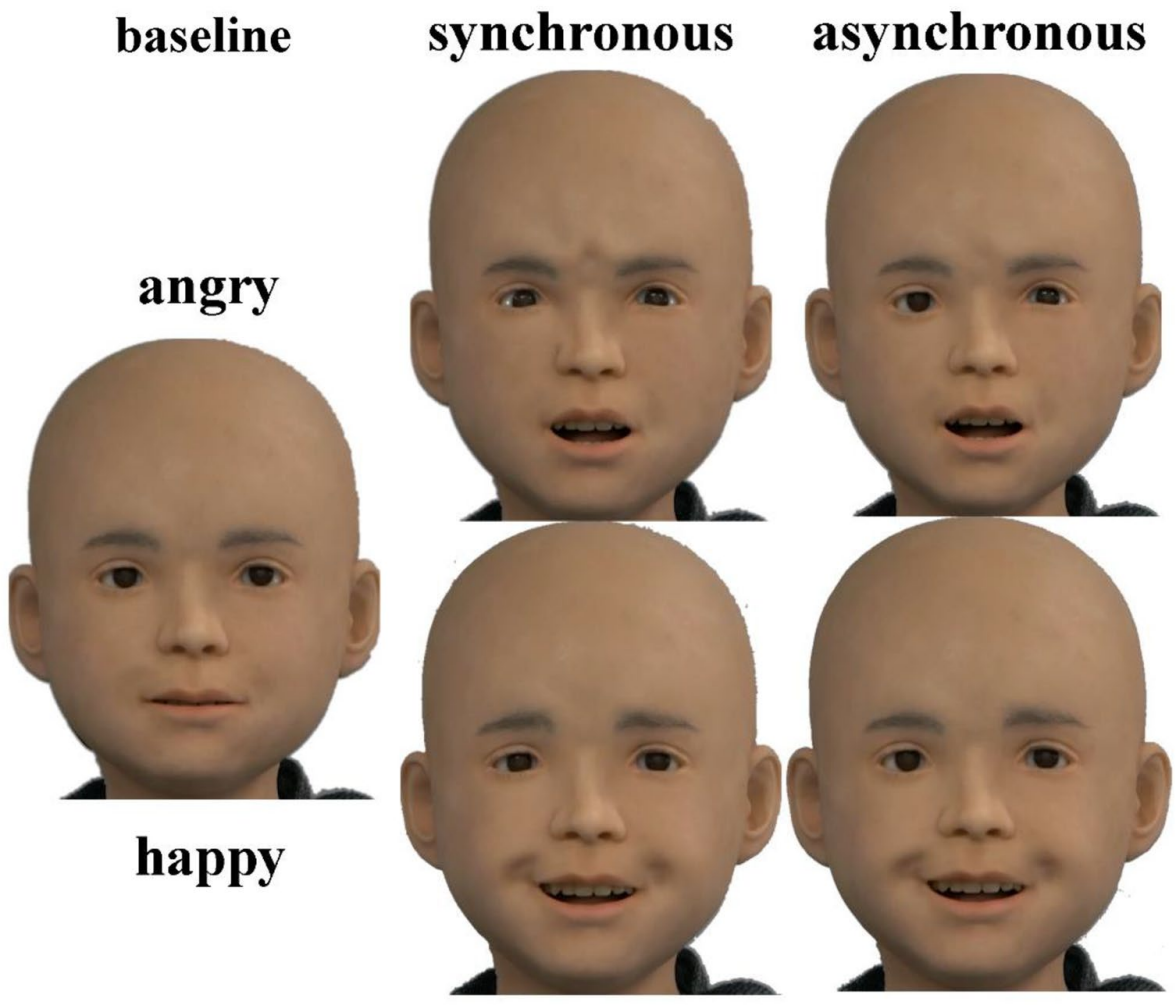
Android expression stimuli divided by condition. *Note*. Baseline (neutral) expression is depicted to the left, followed by synchronous and 500 ms delay asynchronous expressions. The top and bottom rows show angry and happy expressions, respectively.

To control the stimuli, all videos were manipulated to have a white background and to have the agents’ noses at the same height, with cut-offs at the neck (bottom), head (top), and ears (left and right). A total of 16 videos were used in the study. Android stimuli are depicted in Fig. 4. Because the AIST prohibits the distribution of stimulus material due to the risk of public familiarization as a confounding variable, human stimuli are not depicted.

### Stimulus validation

To validate objective and subjective comparability between the android’s and human’s emotion expressions, two analyses were conducted.

#### Objective validation

**Figure 5:**
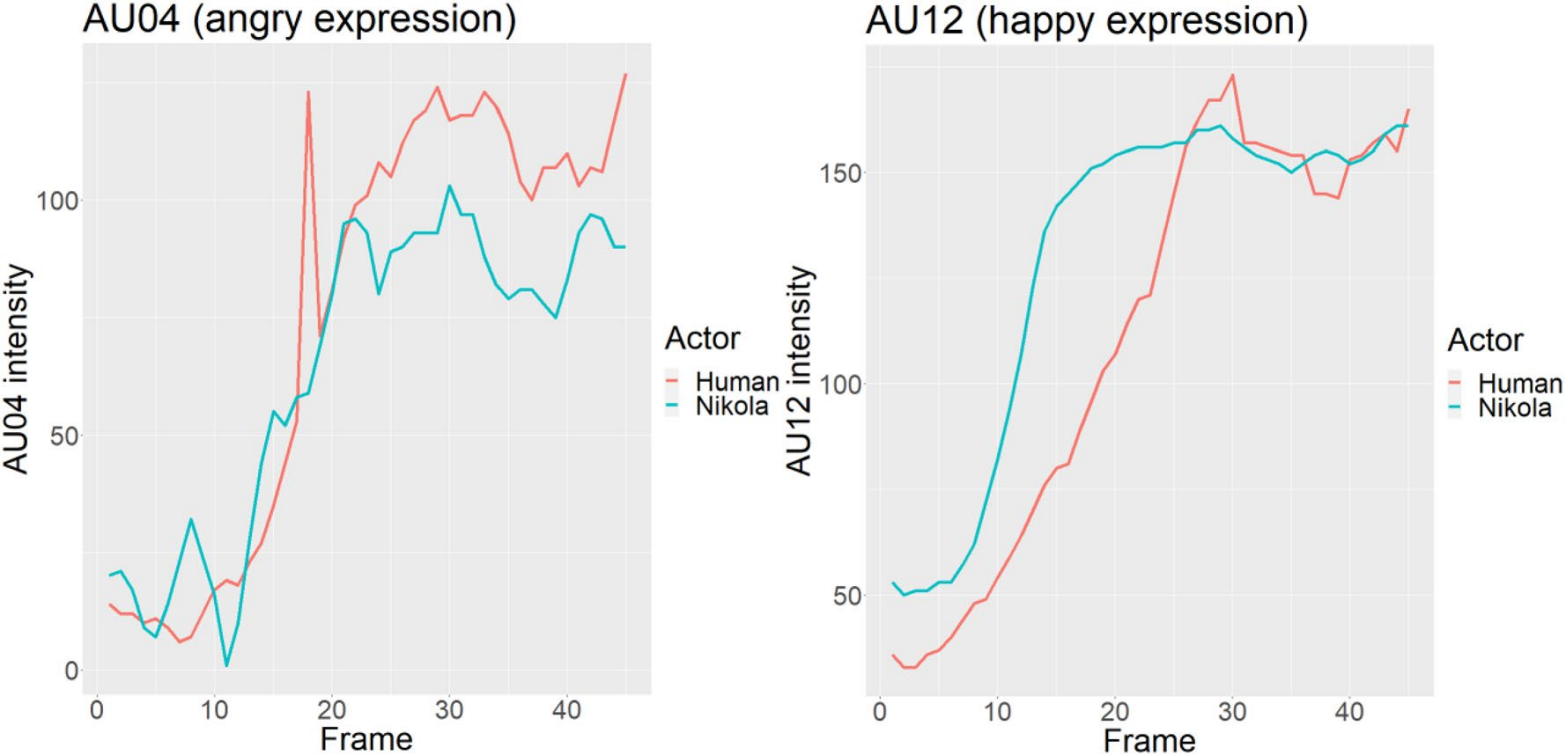
Trajectories of AU04 (brow lowerer) and AU12 (lip corner puller) intensities across video frames, for human and android expressions.

First, facial expressions were analysed using OpenFace (version 2.2.0)^33^. Intensity of face action units (AUs) as indicators for angry and happy expressions respectively over the course of the video are depicted in Fig. 5. For angry expressions, AU4 (brow lowerer) was used, and for happy expressions, AU12 (lip corner puller). Figure 5 indicates analogous trajectories for human and android actors.

#### Subjective validation

An online pilot study (*n* = 11) was conducted using single-scale items of angry and happy recognition as well as arousal and valence, ranging from 0 to 100. Within-subject ANOVAs were conducted with actor type (android, human) as predictors. No significant main effects of actor were found for angry recognition (*F*(1,10) = 1.42, *p* = 0.31), happy recognition (*F*(1,10) = 0.36, *p* = 0.56), arousal (*F*(1,10) > 0.01, *p* = 0.98), or valence (*F*(1,10) = 0.37, *p* = 0.56).

Thus, both objective and subjective validation indicates analogous intensities between android and human expressions.

### Procedure

The study was conducted online. Participants were shown the videos in a randomized order and rated each expression on the scales of *angry, happy*, *arousal*, and *valence* after being given descriptions for each scale. For *angry* and *happy*, participants were told to rate how angry or happy they perceive the expression. For *arousal* and *valence*, participants were told to rate the level of excitement and pleasantness (vs unpleasantness) expressed by the emotions. Each scale ranged from 0 to 100 and participants had unlimited time to freely select a value. Videos were presented on repeat.

### Statistical analysis

The software RStudio® (R version 4.1.2) was used for data preparation and analysis. Analyses of variance (ANOVAs) with orientation (upright and inverted), asynchrony (synchrony and asynchrony), agent type (human and android), and emotion (angry and happy) as within-subject factors were used for emotion recognition (i.e., angry and happy) and dimensional ratings (i.e., valence and arousal). Based on our interest, we conducted follow-up analyses testing the effect of orientation and asynchrony using Tukey’s method.

### Supplementary Information

Supplementary Information.

## Data Availability

The datasets generated and analysed during the current study, the analysis script, and the android expression stimuli are available in the Open Science Framework repository, available at https://osf.io/j247a.
