# Supplementary material for: Differences in configural processing for human versus android dynamic facial expressions
Source: Sci Rep. 2023 Oct 7;13:16952. doi: 10.1038/s41598-023-44140-4 (PMC10560218; doi:10.1038/s41598-023-44140-4)

**Appendix**

**Figure A1**

*Average angry and happy recognition ratings for angry and happy expressions in upright and inverted faces. Error bars indicate standard errors. Post-hoc analyses did not reveal significant differences.*


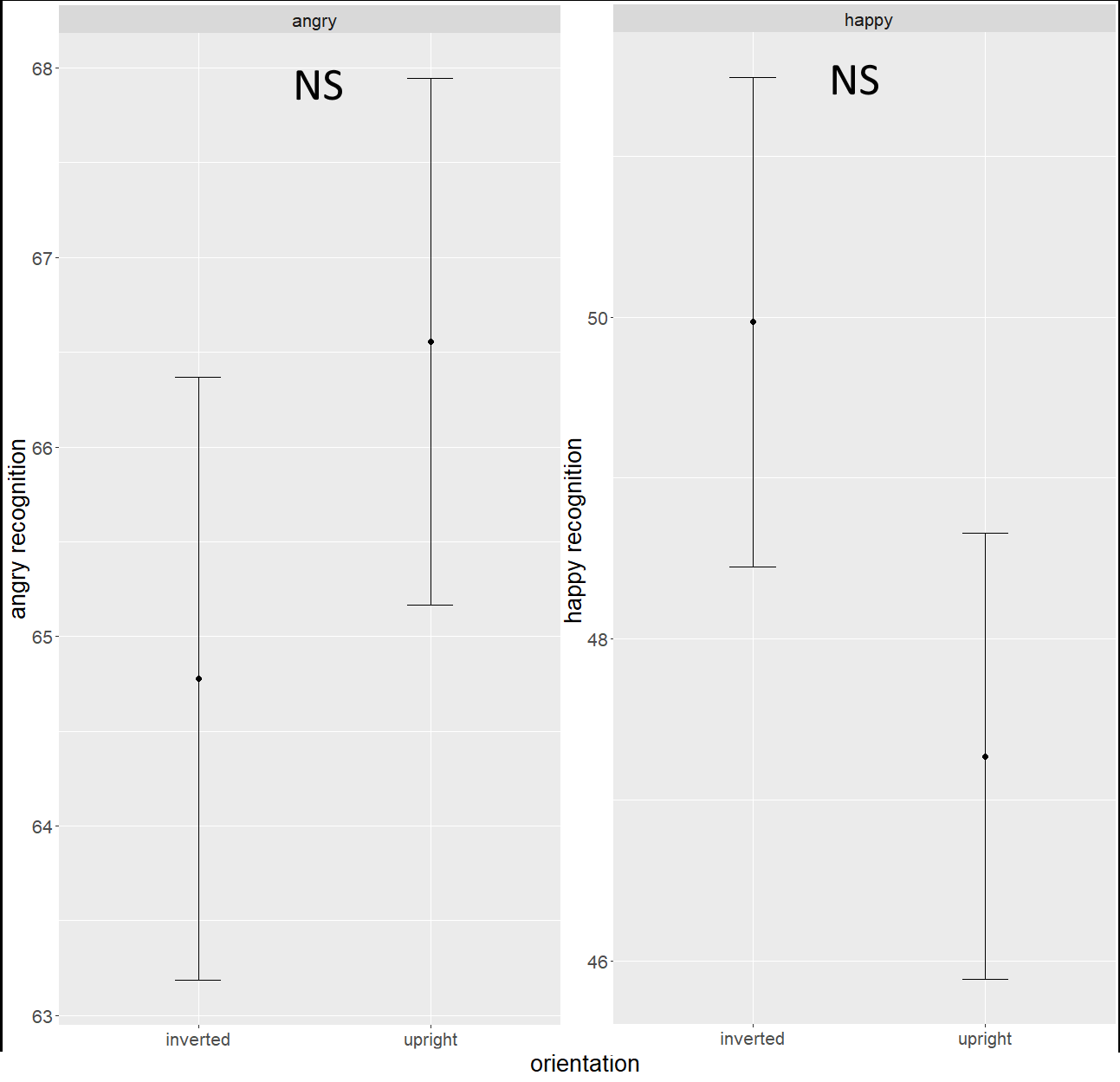

Supplement: Supplementary file 1 — Supplementary Information. [file 41598_2023_44140_MOESM1_ESM.docx]
